# Right Ventricle Strain Assessed by 2-Dimensional Speckle Tracking Echocardiography (2D-STE) to Evaluate Pulmonary Hypertension in Dogs with *Dirofilaria immitis*

**DOI:** 10.3390/ani14010026

**Published:** 2023-12-20

**Authors:** Jorge Isidoro Matos, Sara Nieves García-Rodríguez, Noelia Costa-Rodríguez, Alicia Caro-Vadillo, Elena Carretón, José Alberto Montoya-Alonso

**Affiliations:** 1 Internal Medicine, Faculty of Veterinary Medicine, Research Institute of Biomedical and Health Sciences (IUIBS), University of Las Palmas de Gran Canaria, 35001 Las Palmas de Gran Canaria, Spain; jorge.matos@ulpgc.es (J.I.M.); saranieves.garcia@ulpgc.es (S.N.G.-R.); alberto.montoya@ulpgc.es (J.A.M.-A.); 2 Hospital Clínico Veterinario, Faculty of Veterinary Medicine, Universidad Complutense de Madrid (UCM), 28040 Madrid, Spain

**Keywords:** *Dirofilaria immitis*, heartworm, echocardiography, speckle tracking, strain, pulmonary hypertension

## Abstract

**Simple Summary:**

The development of new echocardiographic techniques, such as two-dimensional speckle tracking echocardiography and assessment of longitudinal myocardial deformation, may be new alternative tools to analyse the presence and severity of pulmonary hypertension in heartworm disease. A total of 93 dogs were used, of which 71% were diagnosed with heartworm infection and 41% were found to have PH. The measurements evaluated were obtained using Right Ventricular Automated Function Imaging (RV AFI^®^) software. The results showed significant differences between animals with and without pulmonary hypertension. Cut-off values with high sensitivity and specificity were also obtained for the detection of pulmonary hypertension in the animals analysed. Echocardiographic measurements for functional assessment of the right ventricle using myocardial longitudinal strain have demonstrated their usefulness in heartworm-infected dogs.

**Abstract:**

Echocardiographic assessment of the right ventricle is helpful for analysing the pathophysiology of heartworm disease and detecting pulmonary hypertension (PH) in dogs. In veterinary cardiology, the study of myocardial deformation using two-dimensional speckle tracking (2D-STE) echocardiography has become increasingly acknowledged as useful for quantifying right ventricular function. The aim of this study was to evaluate the usefulness of myocardial deformation strain of the right ventricular free wall (FWS), global deformation strain of the right ventricle, including the interventricular septum (GS), and tissue motion annular displacement of the tricuspid valve (TMAD) in a cohort of dogs with heartworm (*Dirofilaria immitis*) disease and to determine cut-off values for detecting the presence of PH. Out of the 93 dogs tested, 71% were diagnosed with heartworm infection. PH was identified in 41% of the infected dogs following the American College of Veterinary Internal Medicine (ACVIM) guidelines, based on the peak tricuspid regurgitation velocity to calculate the tricuspid regurgitation pressure gradient (TRPG), while other routine measurements were used, including the right pulmonary artery distensibility index (RPADi). The 2D-STE mode measurements were determined using Right Ventricular Automated Function Imaging (RV AFI^®^) software. The statistical analysis showed significant differences in the studied parameters among dogs with and without PH. Additionally, sensitivity (sen) and specificity (sp) cut-off values were obtained (GS ≥ −21.25%, sen 96%, sp 86.4%; FWS ≥ −21.95%, sen 92.56%, sp 95.5%; TMAD ≤ 0.85 cm, sen 70.4%, sp 83.3%). These results demonstrated that GS, FWS, and TMAD could be used as supplementary and alternative variables to conventional echocardiographic measurements when detecting PH in dogs with heartworm disease.

## 1. Background

Canine heartworm disease (*Dirofilaria immitis*) has a major impact on the cardiovascular system of infected animals, primarily causing pulmonary endarteritis in all and chronic and irreversible precapillary pulmonary hypertension (PH) in some dogs [1,2]. The increase in pulmonary vascular resistance results in a pressure overload in the right ventricle, which can lead to systolic or diastolic dysfunction and right congestive heart failure (R-CHF) [2].

The contractile functionality of the right ventricle is difficult to assess due to its unique anatomical and functional characteristics [3]. Several studies have confirmed that the right ventricle contracts centripetally, with each myocardial segment moving towards the centre. In addition, the base and apex rotate in opposite directions while the ventricle shortens along the longitudinal axis [4,5]. Echocardiographic two-dimensional speckle tracking (2D-STE) allows the analysis of the percentage change in length or thickness of a myocardial segment during the cardiac cycle, which is called myocardial deformation or strain [6]. The free wall of the right ventricle has predominantly longitudinal fibres and, to a lesser extent, circumferential fibres, whereas the interventricular septum has crossed fibres coming from the left ventricular segments and shows a different deformation behaviour. Therefore, analysis of the right ventricular free wall has been considered more specific to the intrinsic function of the right ventricle [7]. However, evaluation of the global functionality of the entire right ventricular myocardium, including the interventricular septum, is commonly used in cardiology [6,7,8].

Longitudinal strain, as measured by 2D-STE, provides an opportunity to quantify both the magnitude and timing of regional, systolic, and diastolic function in specific segments of the myocardium [8]. Many echocardiographic studies have assessed myocardial deformation of the right ventricular free wall (FWS) and global deformation of the right ventricle, including the interventricular septum (GS), in humans [3,4,5]. In veterinary studies, FWS and GS have been considered normal in healthy dogs when less than −20.8% [9,10,11,12] and −18.35% [11,12], respectively. In general, more negative strain values express better systolic function.

Many publications have confirmed the value and clinical utility of right ventricular strain in the study of PH, either independently or in addition to conventional clinical and echocardiographic parameters. In human medicine, a significant correlation has been found between GS and FWS and increased mortality in patients with PH, demonstrating prognostic value [3,4,5]. Recently, in veterinary practice, several studies have reported FWS and GS values in dogs with precapillary and postcapillary PH caused by different diseases [13,14,15,16].

Recently, the use of tissue motion annular displacement of the tricuspid valve (TMAD) has been reported as a measure to study systolic function obtained by echocardiographic study in 2D-STE mode in pets [17]. This parameter evaluates the displacement of the valve annulus towards the apex of the heart, and several studies have shown an adequate correlation of this echocardiographic determination with respect to ventricular function with the tricuspid valve in healthy dogs [18,19,20]. In addition, significantly lower TMAD values have been reported in humans with PH compared to healthy individuals [3].

Due to the high prevalence of PH in dogs with heartworm disease and the difficulty of detecting it echocardiographically in some dogs, it would be prudent to investigate if right ventricular longitudinal strain and tricuspid annular displacement are measurements that might be useful for helping to detect PH in these animals. Therefore, the aim of this study was to evaluate the usefulness of GS, FWS, and TMAD measurements in *D. immitis*-infected dogs and to determine cut-off values for detecting the presence of PH.

## 2. Materials and Methods

### 2.1. Animals

A convenience sample of 93 privately owned dogs was prospectively analysed. The tests were performed at the Veterinary Teaching Hospital of the University of Las Palmas de Gran Canaria (Canary Islands, Spain) between September 2022 and April 2023 during the routine examination of canine patients prior to treatment for heartworm disease or as regular controls in healthy animals. All dogs lived in a hyperendemic area for *D. immitis* [21]. A complete record was kept of each animal, including identification (age, sex, breed, and weight), clinical history, and demographic data. In addition, all were carefully examined for the presence of R-CHF (i.e., one or more of these signs: ascites, pleural effusion, jugular pulse, and/or vena cava distension). A commercial immunochromatography test kit (Urano test Dirofilaria^®^, Urano Vet SL, Barcelona, Spain) was used to determine the presence or absence of *D. immitis* antigen (94.4% sensitivity, 100% specificity). Control dogs were considered free of the disease on the basis of their clinical history (healthy animals, good owner compliance with chemoprophylaxis, dogs are on heartworm prevention year-round), clinical examination, absence of heartworm antigen, no visualisation of worms by echocardiography, and no radiographic evidence of heartworm disease.

Any animal receiving cardiovascular medication was excluded from the study. Similarly, dogs with clinical signs of heart disease (i.e., valvular heart disease, cardiomyopathy, and congenital defects) were excluded. The presence of other respiratory diseases that could cause precapillary PH was also excluded by radiological examination [1].

### 2.2. Conventional Echocardiography

Echocardiographic examinations were performed using an ultrasound machine with spectral and colour Doppler and multifrequency phased array transducers (2.5–12 MHz, Vivid Iq^®^, General Electric, Boston, MA, USA). Examinations were performed with the animals conscious and without the use of sedation. Dogs were placed in right and left lateral recumbency, with the transducer placed in the third-fifth intercostal space, according to previously described techniques [22]. Electrocardiographic monitoring was performed in all cases, allowing assessment of heart rate by measurement of the R–R interval. An average of 3 consecutive cardiac cycles in sinus rhythm was used for each measurement. All echocardiographic examinations were performed by the same researcher.

The presence or absence of PH was determined according to the American College of Veterinary Internal Medicine (ACVIM) guidelines [1]. The peak tricuspid regurgitation velocity to calculate the tricuspid regurgitation pressure gradient (TRPG) was the main measurement, while other routine measurements were used as previously described [1,23,24,25], such as the right pulmonary artery distensibility index (RPADi), the ratio of the pulmonary trunk to the ascending aorta in the right parasternal short-axis view (PT:Ao), the systolic displacement of the tricuspid annular muscle using a one-dimensional mode (TAPSE), and the maximum myocardial velocity (S’) measured in the lateral portion of the tricuspid annulus by tissue Doppler imaging [26,27,28]. The RPADi results were used to evaluate the results, based on previous studies in dogs with heartworm disease and PH [23,24], which demonstrated strong correlation with invasive “gold standard” systolic pulmonary artery pressures.

Relative parasite burden was assessed based on the score published by Venco et al., 2014 [29], which ranges from 1 to 4:1, no worms visualised; 2, few compatible images in the distal part of the right pulmonary artery; 3, compatible images in the right pulmonary artery extending to the main pulmonary artery; and 4, images of worms occupying the entire right pulmonary artery and the main pulmonary artery to the level of the pulmonary valve.

### 2.3. 2D-STE Echocardiography 

Two-dimensional strain measurements were obtained using the same ultrasound equipment, from a left apical 4-chamber view optimised for adequate visualisation of the right chambers of the heart [22]. All studies achieved optimal frame rates > 40 frames per second. Images from all examinations were analysed using Right Ventricle Automated Function Imaging (RV AFI^®^) software (General Electric, Boston, MA, USA).

**Figure 1:**
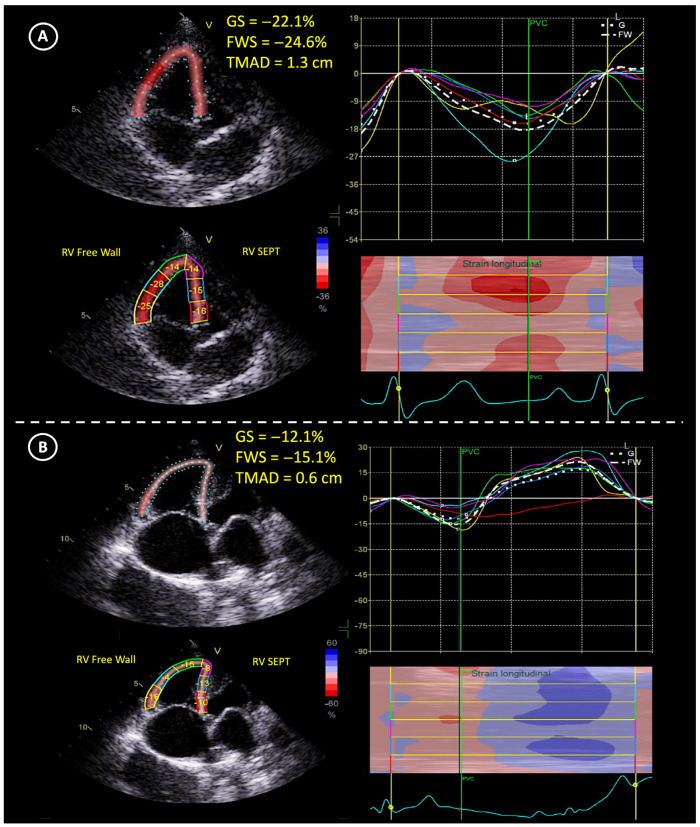
Representation of the analysis of the right strain through the RV AFI^®^ software to obtain the values of GS (%), FWS (%), and TMAD (cm) in (**A**) a patient with heartworm disease without PH, and (**B**) in a patient with heartworm disease that presented PH (RPADi < 30%). Left parasternal position, left apical four-chamber view with >40 frames per second. The graphs show the movement and deformation of each of the myocardial segments in specific colours during the cardiac cycle (apical FW, mean FW, reference FW, apical IVS, mean IVS, baseline IVS). The mean FWS value is represented by a segmented white line, and the mean GS value is represented by a white dotted line. Legend: PVC: peak ventricular contraction; RV: right ventricle.

RV AFI^®^ software used spot-tracking technology to quantify specific right ventricular myocardial motion, with some custom adaptations to better capture right ventricular free wall motion. The region of interest was defined by the operator to include the entire right ventricular myocardium and the interventricular septum. The operator placed two points on the parietal and septal annuli of the tricuspid valve and one point on the right ventricular apex, starting with end-systole and followed by end-diastole. The system then automatically provided an estimate of the area of interest, which could be regionally edited if desired. RV AFI^®^ software tracked the acquired image over time, extracting information on regional and longitudinal strain. The software provided segmental longitudinal strain values for the following 6 segments: apical FW, mid FW, baseline FW, apical IVS, mid IVS, baseline IVS. Finally, the results were provided, showing the quality of the follow-up and the values in percentage of GS, percentage of FWS, and centimetres of TMAD (Figure 1).

### 2.4. Statistical Analysis

Statistical analysis of echocardiographic values was performed using commercially available software (BM SPSS Statistics 25.0, New York, NY, USA). Frequencies and percentages were calculated for categorical variables. Parameter differences between groups were assessed using Pearson’s non-parametric Chi-squared test and, only in the case of 2 × 2 tables, Fisher’s exact test. For continuous variables, parameter differences between groups were evaluated using the Mann–Whitney/Kruskal–Wallis test (non-parametric) or t-student/ANOVA (parametric), depending on the normality of the variables to be evaluated, as determined by the Shapiro–Wilk test. Linear regressions were used to determine the correlation and explanatory power of the methods with the gold standard (RPADi), and the non-standardised beta coefficients, their 95% CIs, R2, and equations were presented. The receiver operating characteristic (ROC) curve was used to determine the ability of the different echocardiographic measurements to classify the presence/absence of PH. All multiple comparisons were adjusted by Bonferroni correction. All contrasts were accompanied by an effect size estimate to complete the interpretation of the results. For categorical variables, this was Cramer’s V, and for continuous variables, Cohen’s d. The criteria for classifying the size of the effect were as follows: Cohen’s d: small (d = 0.2–0.4), medium (d = 0.5–0.8), and large (d = greater than 0.8). Cramer’s V: 0.00–0.09 as negligible, 0.10–0.29 as low, 0.30–0.49 as medium, and from 0.50 as high. In the case of effect size estimator values obtained from inversely correlated variables, the results were expressed as negative (−). In all cases, the significance level used was 5% (α = 0.05).

### 2.5. Ethical Statement 

All owners were informed and gave consent for their dogs to be included in the study. Ethical approval was not required for this study as it was a purely observational study with voluntary enrolment and did not involve any additional invasive clinical diagnostic procedures. The evaluation of the study included ethical considerations and legal aspects of animal protection and welfare and was carried out in accordance with current Spanish and European legislation on animal protection. 

## 3. Results

Based on the immunochromatographic test kit, 71% (*n* = 66) of the dogs were infected with *D. immitis*, and 29% (*n* = 27) were considered healthy based on the absence of *D. immitis* antigens, clinical signs, history, physical examination, and echocardiographic and radiographic evaluations.

Following the ACVIM consensus criteria [1], PH was present in 41% (*n* = 27) of heartworm-infected dogs, whereas PH was absent in all healthy dogs. According to heartworm status and the presence of PH, three groups were created: group A (healthy dogs, *n* = 27), group B (heartworm-infected dogs without PH, *n* = 39), and group C (dogs with heartworm and PH, *n* = 27). All dogs with PH showed an RPADi < 30%. The clinical characteristics, conventional echocardiographic parameters, and 2D-STE echocardiographic measurements of the total animal population and in each of the different groups are shown in Table 1. 

A wide age range (2–15 years) was present in the dogs studied, both sexes were present in similar proportions (51.6% females and 48.4% males), and weights ranged from 3.2 to 45.9 kg. Twenty-four different breeds were included in the study, with mixed breeds being the most common (45.2%). There were no significant differences in age, sex, breed, or weight between the groups. 

The observed parasite burden score ranged from 1 to 4 in dogs with heartworm. A higher burden was observed in dogs from group C compared to group B (*p* Chi2 value < 0.01; Cramer’s V = 0.841). The relative parasite burden in healthy dogs was 1 (no visible worms). The signs of R-CHF were directly related to the presence of heartworm and PH (*p* Chi2 value < 0.01; Cramer’s V = 0.602), as signs of R-CHF were present only in group C (44.4%).

All conventional echocardiographic measurements showed significant differences in group C when compared to groups A and B (*p*-value ANOVA < 0.01; Cohen’s d > 1.00). The results showed higher values for TRPG and PT:Ao and lower values for TAPSE and S’ in group C. No significant differences were observed between the animals in groups A and B in any of the parameters evaluated.

**Figure 2:**
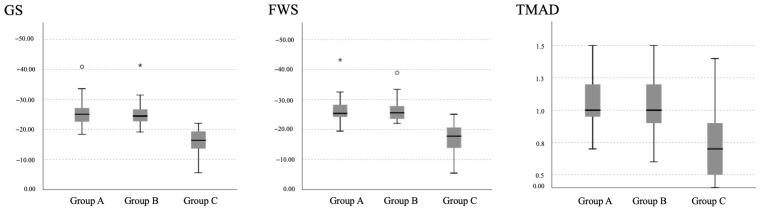
Echocardiographic 2D-STE measurements of GS (%), FWS (%), and TMAD (cm) obtained in healthy dogs (group A), dogs with heartworm without PH (group B), and dogs with heartworm with PH (group C). Box plots show median (solid horizontal lines within boxes), 25th and 75th percentiles (boxes), and minimum and maximum values (whiskers). Statistically significant differences were observed in group C when compared to groups A and B in all cases. Legend: white circle = outlier; asterisk = extreme outlier.

The ANOVA tests of the parameters obtained by 2D-STE indicate that there were significant differences in the values of GS, FWS, and TMAD between the groups studied (Figure 2). Bonferroni’s post hoc tests for multiple comparisons showed that the values obtained in dogs from group C were significantly lower than those obtained in groups A and B (*p*-value ANOVA < 0.01; Cohen’s d > 1.00), whereas groups A and B did not differ from each other.

**Figure 3:**
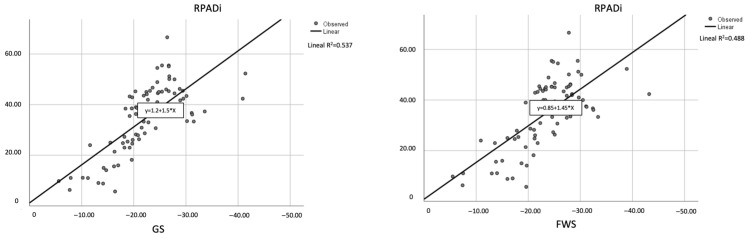
Scatter plots showing significant (both *p* < 0.01) coefficient of determination (R^2^) between RPADi and 2D-STE measurements (GS and FWS). The solid line within each scatterplot represents the line of best fit.

A total of three linear regressions were performed, one for each 2D-STE parameter, to determine which variables correlated best with the RPADi. Pearson’s correlations showed values of 0.733, 0.698, and 0.555 for the variables of GS, FWS, and TMAD, respectively (Figure 3). The measurements that correlated best with the RPADi were GS (R^2^ = 0.537), followed by FWS (R^2^ = 0.488) and TMAD (R^2^ = 0.300). All models were calculated in 93 cases. 

The results of the ROC curves for each of the 2D-STE measurements to determine the presence or absence of PH and the cut-off points of the parameters that maximised sensitivity and specificity (using the Youden index) are summarised in Table 2. GS and FWS showed an excellent area under the curve (AUC) (>0.9), while TMAD had an optimal AUC of 0.830. To detect PH, the cut-off values were ≥−21.25% for GS (sensitivity 96.3%; specificity 86.4%), ≥−21.95% for FWS (sensitivity 92.6%; specificity 95.5%), and ≤0.85 cm for TMAD (sensitivity 70.4%; specificity 83.3%).

## 4. Discussion

The presence of heartworms in the pulmonary arteries causes endarteritis and embolization that leads to a loss of vascular elasticity and an increase in vascular resistance, which, when severe, leads to pre-capillary PH [30]. This produces an increase in right ventricular afterload, which leads to a decrease in ventricular contraction (strain). PH is common in dogs with heartworm disease (although it is often not present), occurring in 30–67% of infected dogs (41% in the current study) [2,23,24,28]. When severe, PH can lead to R-CHF [2,28]. 

Previous studies have found no evidence that sex, breed, weight, or age are risk factors for *D. immitis* infection [27,28,29,30]. In the present study, dogs with PH had a higher relative worm burden. While some previous studies have agreed with this assessment, others have found disease chronicity, exercise, and host immune response to be more important factors [2,23,24,28,29,30]. 

The employment of RPADi has been ratified by numerous studies as a useful method to help in the detection of PH in dogs infected with *D. immitis*. In this study, PH was identified in 41% of dogs with heartworm, and in all cases, RPADi was <30% [23,24,28,30]. Additionally, previous studies have demonstrated statistically significant differences in dogs with PH compared to those without, regardless of whether they were healthy or infected with *D. immitis* [1,2,8,9,10,11,12].

Speckle tracking is a technique used in echocardiography to assess myocardial strain, which refers to the deformation (contraction and relaxation) of the heart muscle during the cardiac cycle. Speckle tracking involves tracking the movement of small, natural acoustic markers (speckles) within the myocardial tissue. These speckles appear as small, bright dots in the ultrasound images. Specialised software is used to identify and track these speckles frame by frame throughout the cardiac cycle. As the heart contracts and relaxes, the speckle-tracking software analyses the displacement of the speckles in the myocardial tissue. The software calculates strain, which is a measure of the percentage change in length of the myocardial segments during contraction or relaxation. Much like fractional shortening, systolic strain is a measure of the percent change in the length of myocardial segments in systole. As opposed to fractional shortening, which only measures global shortening, strain can measure both global and regional functions [11]. While several studies have used strain to examine left ventricular function in dogs with left heart diseases [7,14,15,26,31], measuring strain in the right ventricle is particularly advantageous because of the unusual shape of the right ventricle. 

The complex morphology of the right side of the heart makes the analysis of right ventricular function difficult. Blood flows are complicated to quantify in a simple form, and the use of tissue Doppler provides limited information on systolic and diastolic function. Compared to conventional echocardiographic techniques, 2D STE evaluates longitudinal and circumferential right ventricular myocardial deformation (influenced by contractility, preload, and afterload) very efficiently and accurately [26,27]. The values obtained in animals without PH were similar to those previously reported in healthy dogs, which were values <−24.85% for GS and <−26.12% for FWS, in addition to values >0.95 cm for TMAD [11,13,14,15,16,19,22]. 

Significant statistical differences were observed in the values of GS, FWS, and TMAD between dogs with PH and normotensive dogs. This suggests that these variables may be useful in helping to diagnose PH in dogs with heartworm disease. The established values and cut-off points for GS and FWS in this study align with prior research on dogs with precapillary PH due to various diseases [9,13]. However, the GS and FWS measurements were slightly different when compared to previous studies. This may be attributed to methodological differences, including sample size, the type of 2D-STE software used, and the specific diseases assessed (mainly myxomatous mitral valve disease) [7,8,9,10,11,12,13,14]. Interestingly, previous studies also found lower FWS values when compared to GS [12,13,14,15]. This may be due to the right ventricular free wall being the key component of right ventricular functionality. In contrast, circumferential strain measurements showed higher values in patients with various causes of PH, as reported by Caivano et al. (2020) [16]. The transverse strain of the right ventricle in our study did not aid in identifying the presence or absence of PH, suggesting that longitudinal measurements may be more sensitive for this diagnosis. No prior studies have recorded TMAD in dogs with PH. Our results suggest a cut-off value of ≤0.85 cm for detecting the presence of PH in dogs with heartworm disease.

In the present study, the GS method displayed the best correlation with the RPADi; however, FWS was the parameter with the highest sensitivity and specificity for detecting PH in the dogs analysed. This study’s findings align with previous publications that demonstrated the higher specificity and sensitivity of FWS compared to GS for the detection of PH in both dogs and humans [3,4,5,9,13]. On the other hand, despite exhibiting a reasonable AUC, the TMAD measurement was the least helpful parameter in diagnosing PH in heartworm-infected dogs.

The high cost of specialised software is a key drawback to the use of 2D-STE echocardiography in veterinary cardiology. Furthermore, identification of the appropriate echocardiographic view, which should be optimised to facilitate the visualisation of the right chambers whilst maintaining high frames per second and good resolution, is crucial. However, 2D-STE studies are less sensitive to angle limitations compared to other traditional measurements, leading to more reliable outcomes and decreased intra- and inter-operator deviation [7,10,14]. Furthermore, it is acknowledged as an ultrasound imaging mode characterised by good reproducibility, although in certain studies, body weight, heart rate, and age have reportedly influenced measurements, which has not been documented in the present research [9,11,12,18]. The unique diagnostic value of being able to identify abnormalities in each myocardial segment sets this echocardiographic technique apart from others. However, caution must be exercised in interpreting the results, as they depend on the analysis software used.

## 5. Conclusions

This study demonstrated that measurements of right ventricular longitudinal strain may be useful to assist in the detection of PH in dogs with *D. immitis*. These measurements may be useful as additional or alternative measurements for the detection of PH alongside traditional echocardiographic measurements. This study has provided cut-off points for detecting PH in dogs with heartworm disease with optimal sensitivity and specificity. However, further research is required to validate these results.

## Figures and Tables

**Table 1:** Clinical and echocardiographic parameters of the dogs studied (*n* = 93). Data represent median and standard deviation, unless otherwise indicated. Group A: healthy dogs; group B: dogs with heartworms and absence of PH; group C: dogs with heartworms and with PH (RPADi < 30%). Legend: CHF: congestive heart failure; HR: heart rate; RPADi: right pulmonary artery distensibility index; TRPG: tricuspid regurgitation pressure gradient; PT: pulmonary trunk; Ao: aorta; TAPSE: systolic displacement of the tricuspid annular muscle; S: maximum myocardial velocity; GS: global strain; FWS: free-wall strain; TMAD: tissue motion annular displacement of the tricuspid valve. Results for female, mongrel, and right-sided CHF symptoms are expressed as *n* (%). Parasite burden scores and TRPG are expressed as median (range). Significant differences were found when *p* < 0.01.

	Group A (*n* = 27)	Group B (*n* = 39)	Group C (*n* = 27)	*p*-Value (^4^ Cramer’s V; ^5,6^ Cohen’s d)
Age (years)	8.0 ± 3.9	6.8 ± 2.8	8.3 ± 3.2	0.164 ^1^
Female: number (%)	15 (55.6%)	24 (61.5%)	9 (33.3%)	0.07 ^2^
Body weight (kg)	15.6 ± 10.2	19.5 ± 11.1	19.6 ± 9.7	0.51 ^1^
Mongrel: number (%)	9 (33.3%)	20 (51.3%)	13 (48.1%)	0.02 (0.60) ^4^
Right-sided CHF (%)	0 (0%)	0 (0.0%)	12 (44.4%)	0.00 (0.60) ^4^
Parasite burden score (1–4)	1	2 (1–3)	3 (2–4)	0.00 (0.72) ^4^
HR (Beats per minute)	134.4 ± 25.1	131.4 ± 28.1	129.2 ± 21.8	0.871 ^3^
RPADi (%)	42.2 ± 4.7	41.4 ± 8.0	18.8 ± 7.6	0.00 (1.79) ^5^
TRPG (mmHg)	4.49 (2.43–7.07)	3.5 (2.10–5.23)	49.44 (38.21–152.15)	0.00 (−1.32) ^5^
PT:Ao	1.0 ± 0.1	0.9 ± 0.1	1.3 ± 0.2	0.00 (−1.47) ^5^
TAPSE (cm)	1.7 ± 0.3	1.6 ± 0.3	1.1 ± 0.3	0.00 (1.62) ^5^
S (cm/s)	14.7 ± 2.5	15.7 ± 3.4	9.3 ± 3.8	0.00 (1.08) ^6^
TMAD (cm)	1.1 ± 0.2	1.0 ± 0.2	0.7 ± 0.3	0.00 (1.27) ^6^
GS (%)	−25.6 ± 4.9	−25.2 ± 4.0	−15.8 ± 4.4	0.00 (1.58) ^6^
FWS (%)	−26.7 ± 4.7	−26.2 ± 3.5	−16.9 ± 5.0	0.00 (1.6) ^6^

^1^ Mann–Whitney/Kruskal–Wallis. ^2^ Chi2 test. ^3^ ANOVA. ^4^ Chi2 test = *p* < 0.01 (Cramer’s V in parentheses). ^5^ Mann–Whitney/Kruskal–Wallis = *p* < 0.01 (Cohen’s d value in parentheses). ^6^ ANOVA = *p* < 0.01 (Cohen’s d value in parentheses).

**Table 2:** Results of simple regression analyses for the prediction of RPADi (R^2^: coefficient of determination). Therefore, sensitivity (Se), specificity (Sp), and Youden index of cut-off points of echocardiographic 2D-STE measurements for predicting PH for detecting RPADi < 30%. AUC: area under the receiver operating characteristic curve; CI 95%: confidence interval 95%.

	R^2^	AUC	IC 95%	Cut-off	Se	Sp	Youden Index (Se + Es − 1)
FWS (%)	0.482	0.962	(0.917, 1.000)	≤21.95	0.926	0.955	0.880
GS (%)	0.532	0.970	(0.943, 0.998)	≥−21.25	0.963	0.864	0.827
TMAD (cm)	0.300	0.830	(0.725, 0.935)	≤0.850	0.704	0.833	0.537

## Data Availability

The raw data supporting the conclusions of this article will be made available by the authors, without undue reservation.
